# Perceptions and Practices of the Iranian Population regarding Skin Cancers: A Literature Review

**DOI:** 10.1155/2017/4934108

**Published:** 2017-11-19

**Authors:** Vinayak K. Nahar, Zachria Hasani, Brian Martin, Javier F. Boyas, Rosa Chabok, Leena S. Philip, Ghazal Ghafari, Leila Seidfaraji, Stacy Chelf, Ram Lakhan, Amanda H. Wilkerson, Marcelle Savoy, Manoj Sharma

**Affiliations:** ^1^Center for Animal and Human Health in Appalachia, College of Veterinary Medicine, DeBusk College of Osteopathic Medicine, and School of Mathematics and Sciences, Lincoln Memorial University, Harrogate, TN, USA; ^2^School of Mathematics and Sciences, Lincoln Memorial University, Harrogate, TN, USA; ^3^Department of Social Work, School of Applied Sciences, University of Mississippi, Oxford, MS, USA; ^4^Behavioral & Environmental Health, School of Public Health, Jackson State University, Jackson, MS, USA; ^5^Department of Anatomy, DeBusk College of Osteopathic Medicine, Lincoln Memorial University, Harrogate, TN, USA; ^6^Department of Health and Human Performance, Berea College, Berea, KY, USA; ^7^Department of Health and Exercise Science, College of Arts and Sciences, The University of Oklahoma, Norman, OK, USA; ^8^Dr. Lon and Elizabeth Parr Reed Health Sciences Library, DeBusk College of Osteopathic Medicine, Lincoln Memorial University, Harrogate, TN, USA

## Abstract

Despite being preventable, more than 15% of all cancer cases in Iran occur in the skin, making them the most commonly diagnosed malignancy in the country. The purpose of this study is to gain an insight into the current skin cancer related knowledge, attitudes, beliefs, and practices among the Iranian population. A systematic computer based literature search was conducted using databases for articles published through April 2017. Research studies included those that measured skin cancer or sun protection related knowledge, attitudes, beliefs, and behaviors in different Iranian population groups. Exclusion criteria for the articles included (1) irrelevant topics to the review article's aim, (2) articles that focused on the treatment of skin cancers instead of prevention practices, and (3) similar studies conducted on populations not indigenous to Iran. A total of 25 articles that met the eligibility criteria were included in the review. Predominant data were collected via questionnaires. Skin cancer related knowledge varied from low to high across the studies. Moreover, there was a pattern of low perceived skin cancer susceptibility and severity. Overall, there was low usage of sun protection methods among the Iranian population. The findings of this study show that efforts to prevent skin cancer are needed. Education concerning the dangers of sun exposure as well as strategies used to prevent or lower the risk of developing skin cancer should be stressed.

## 1. Introduction

The World Health Organization (WHO) projects an 80–100% increase in the cancer related mortality rate of individuals residing in east Mediterranean countries over the next 15 years [1]. Despite being preventable, more than 15% of all cancer cases in Iran occur in the skin, making them the most commonly diagnosed malignancy in the country [2, 3]. From 1995 to 2004, the mortality rate of skin cancers in the Iranian population increased significantly from 0.06 to 0.70 per 100,000 people [4]. During this time frame, the male population exhibited an increase in mortality rate from 0.06 to 0.90 per 100,000 people, while the female population's mortality rate increased from 0.05 to 0.48 per 100,000 people [4]. The likelihood also increased with age, particularly among those aged 50 and greater [5]. A study looking at the prevalence of the types of skin cancers in the Iranian province of Yazd revealed that basal cell carcinoma accounted for 76.9% of skin cancer diagnoses, 18.1% was attributed to squamous cell carcinoma, while 2.7% of skin cancer cases in the study were determined to be melanomas [6]. While melanomas are considered the most dangerous type of skin cancer, basal cell carcinomas are the most numerous [5]. It should be noted that both of these types of skin cancers occurring on areas of the face, head, and neck can be attributed to extended sun exposure over a lifetime, as well as acute and intense exposure that induces sunburn [5].

Since 92% of skin cancers develop on the face, head, and neck [6], primary preventive measures can be used to avoid the negative impact of ultraviolet rays. Research on the prevention of skin cancer has indicated a 78% reduction in the lifetime incidence rate of nonmelanoma carcinomas if a sunscreen is applied appropriately from birth to the age of eighteen [7]. Moreover, the American Cancer Society (ACS) cites that additional important preventive methods include using hats and sunglasses, seeking shade between 10:00 a.m. and 4:00 p.m., and wearing clothing that covers as much skin as possible [8]. Material should be dark-colored and should consist of tightly woven fabric. Long pants, skirts, and long-sleeved shirts are ideal [8].

The purpose of this study was to gain an insight into the current skin cancer related knowledge, attitudes, beliefs, and practices among the Iranian people. By evaluating each of these components, it will be possible to identify the best practices among the Iranian population and establish efficient measures that will decrease the prevalence of skin cancers. After an extensive literature search, no evidence of a similar published review has been found by the authors.

## 2. Methods

In the initial search, a systematic computer based literature search was conducted using, Cumulative Index to Nursing and Allied Health Literature, MEDLINE, PsychInfo, PubMed, ScienceDirect, and Scopus databases. The search was performed in electronic databases using combinations of the following terms: “Iran”, “skin cancer”, “sun protection”, “melanoma”, “knowledge”, “attitudes”, “beliefs”, “perceptions”, “sunscreen”, “prevention”, “practices”, and “behaviors”.

To minimize the probability of missed articles, updated additional searches were conducted in Google Scholar and the Shahid Beheshti University of Medical Sciences databases. Also, a more targeted search within Iranian medical publications currently indexed in PubMed included the following journals: Medical Journal of the Islamic Republic of Iran, Acta Medica Iranica, Iranian Biomedical Journal, Archives of Iranian Medicine, Iranian Red Crescent Medical Journal, and the Iranian Journal of Public Health. Additional search terms—“aware” or “awareness”, “education”, “cutaneous”, and “malignancy”—were also included in the updates.

Results of the initial literature search conducted in August 2016 were reviewed by abstracts and titles with the exclusion of off-topic articles. Analyses of further studies updated in April 2017 were performed by reviewing the reference lists of the articles of interest, along with the additional databases.

The search was not limited by the date of publication or language. Both English and Persian language studies were included. All manuscripts published in peer-reviewed journals, to the best of the reviewers' knowledge, were considered for inclusion in this review. Research studies were included that measured skin cancer or sun protection related knowledge, attitudes, beliefs, and behaviors in Iran. Exclusion criteria for the articles included (1) irrelevant topics to the review article's aim, (2) articles that focused on the treatment of skin cancers instead of prevention practices, and (3) similar studies conducted on populations not indigenous to Iran. The literature search was conducted by four independent reviewers. Any disagreement regarding inclusion criteria was resolved via discussion until consensus was reached.

Table 1 provides details of the literature review pertaining to skin cancer knowledge, attitudes, and practices among the Iranian population. The first column lists the first author of each corresponding article, as well as the date and location of the study. Column two includes the methodologies used for data collection and sample size (*n*) for each study along with the gender and age of the participants. Extracted data regarding knowledge, attitudes, and beliefs of the participants are found within the third column. If the included study had an experimental design, then only pretest data was extracted. The last column describes the prevention practices of the subjects surveyed in each study.

## 3. Results

**Figure 1:**
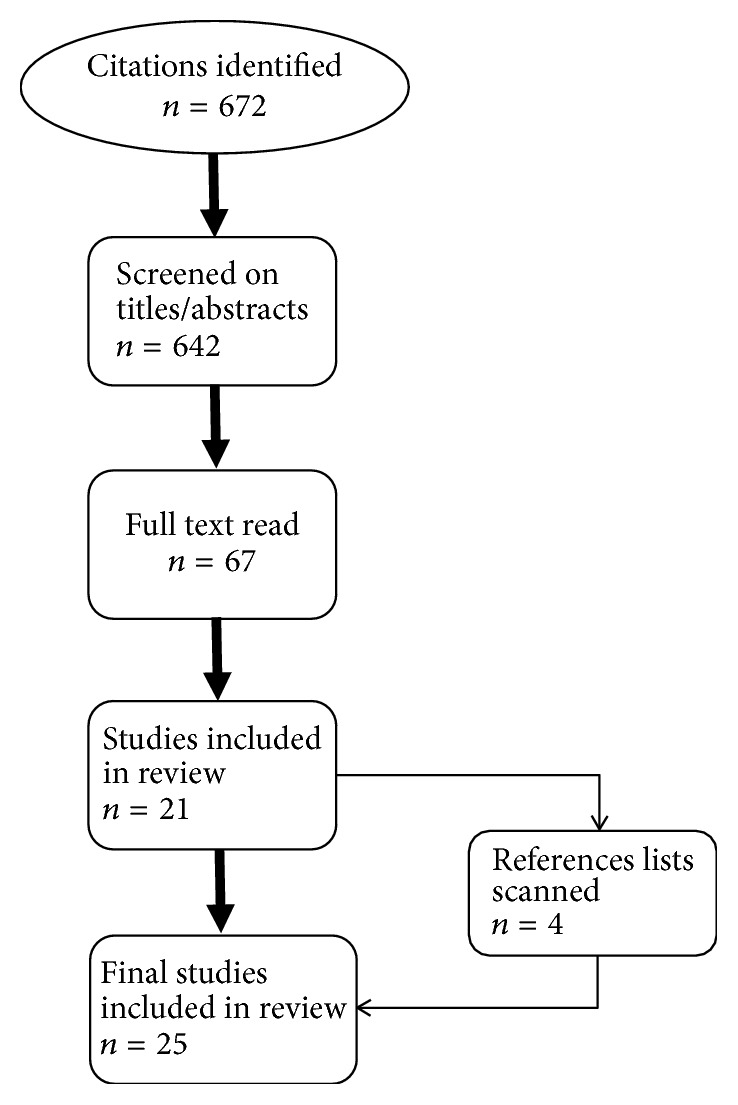
Chart of the literature review.

Electronic searches identified a total of 672 citations. After removing duplicates (*n* = 30), the remaining 642 articles were screened based on titles and abstracts. After screening, the remaining 67 articles were read in their entirety to determine inclusion criteria eligibility. Once the articles were suitably vetted, 21 were selected along with four additional sources identified from their reference lists. In summary, a total of 25 articles met the eligibility criteria and were included in the review (Figure 1) [9–32].

### 3.1. Study Characteristics

The studies were conducted between 2007 and 2016 in various cities across Iran [9–32]. The majority of the data were collected using only questionnaires [10–24, 26–32]. Additionally, two studies conducted interviews along with using questionnaires [9, 25].

### 3.2. Participant Characteristics

The sample size of participants in the studies ranged from 75 to 2,000 [9–32]. There was approximately an even ratio of males to females across studies with some of the studies using only male [22, 28, 31] or female [10, 13, 17, 24, 27, 32] participants. The average ages ranged from 15 to 48 years across the studies that provided this demographic information [9, 10, 12–18, 21–27, 29, 31, 32].

### 3.3. UVR Exposure

In the study conducted by Firooz and colleagues, approximately 61% of the participants reported receiving direct sun exposure between 1 and 4 hours per day [9]. In one study, 88.8% of the participants said they sunbathed <5 times/year and 87.8% never tanned or tanned only once [15]. Another study showed that 24 participants used solarium for tanning [19].

### 3.4. Skin Cancers Related Knowledge, Attitudes, and Beliefs

The participants' skin cancer related knowledge varied across the studies. In one study, 92.3% knew that sunlight prevention practices are important in preventing skin cancer and 57% knew that sunscreens should be used during all seasons [11]. In another study, 67.5% knew the correct application time of the sunscreen; however, only 15% knew the sun protection factor definition perfectly [16]. Tabatabayian et al.'s study reported that 53% of the participants knew about harm from sun exposure, 3.1% had basic knowledge about skin cancer, and 68.5% knew about sun protection methods [26].

Furthermore, a study showed that 72.9% believed that skin cancer was preventable and 20.9% believed that sunbathing can cause cancer [12]. In that same study, 28.2% of the participants agreed that using sun protection methods is difficult and time-consuming, while 13.2% and 25.3% believed that sunscreens and hats/gloves/sunglasses, respectively, are of no use in preventing skin cancer [12]. Another study showed that 58.8% of the participants believed that using sunscreens is not an easy task [15].

### 3.5. Skin Cancer Prevention Practices

In the earliest study, 75% of the participants indicated that they did not practice any sun protection behaviors [11]. In one study, 68.8% indicated sunscreen use, but 72.7% of females and only 27.3% of males used a sunscreen [16]. In another study, of the 52% that used a sunscreen, 80.4% were females and 13.5% were males [18]. This trend proved true for sunglasses usage as well; 35.21% of females and 9.58% of males used sunglasses [23].

Sunglasses use and clothing to protect against UV exposure were less than 50% across the studies [10, 12, 14, 15, 19, 23, 24, 27–30], with gloves having the least amount of use (<15%) [10, 12, 14, 24, 27–30]. Hat usage varied from 1% to 53.5% [10, 12, 14, 24, 27–30].

## 4. Discussion

Skin cancers are highly preventable if protective measures are utilized; yet rates of skin cancer incidence have increased over the years in Iran. The results from this literature review showed that the majority of participants recognized that protecting themselves from sun exposure was important. Many of the participants recognized the harms resulting from prolonged sun exposure; with that being said, the attitude of some indicated that they did not realize the severity of sun exposure. For example, in one of the studies, 54% believed that skin cancers are usually not fatal [12]. In another study involving farmers in rural Iran, only 30.6% had high-perceived severity and 60.1% had an unacceptable threat appraisal of skin cancer [22].

Physicians and health educators in Iran should emphasize the serious risk of sun exposure to their patients to increase the awareness that UV rays are very harmful to their health and may lead to the development of skin cancers. Subjects with different demographics, such as students, farmers, and hospital personnel, were reviewed in this study. Since such varied populations were used, it is recommended that knowledge and awareness concerning how skin cancer specifically targets lifestyle become more prevalent among each specific demographic group. For example, educational activities and lectures may be helpful for students. Other methods include distributing literature and free cancer screenings [33]. It is also critical to emphasize the importance of preventive skin cancer measures. Identifying and educating individuals at an elevated risk, such as those who sunbathe or spend much of their time outdoors, should be a top priority for health professionals. Once identified, education on the risks of sun exposure and applications used to prevent or lower sun exposure should be stressed.

Although the participants in the studies knew the risks of sun exposure, not all participants understood the sun protection application methods, such as how often they should reapply sunscreens. Surprisingly, about 25% of the participants in one study agreed that using sun protection methods was difficult and time-consuming [12]. In a separate study, less than two-thirds believed that preventive measures, such as using sunscreens and clothing, were effective in preventing skin cancers [15]. From the literature, sunscreens were the most prevalent method used to protect against the sun with limited usage of clothing, hats, gloves, and sunglasses. The results suggest that the need for more education on the effectiveness of sun protection methods is crucial. The attitudes and behaviors of participants should also be addressed by informing them of the various methods for sun protection and how to apply these correctly. Overall, skin care prevention practices remain low, suggesting that more emphasis should be placed on linking the impact of sun protective methods to decreased rates of skin cancers.

When addressing the issue of increasing skin cancer prevention practices, it is important to consider both the method of disseminating information and the unique conditions of each group. For example, among rural agricultural communities, men are more likely to be exposed to sunlight than are women. Due to decreased access to medical services, they are also more likely to receive a skin cancer diagnosis at a later stage than someone from an urban community, emphasizing the importance of prevention education [25]. Furthermore, it is important to consider where these individuals are most likely to receive advice about skin cancer prevention. Movaffagh et al. found that, among Iranians, pharmacists constitute a preferred advisory group for questions about sunscreens [16]. This is particularly true of rural and impoverished communities that may believe seeking a doctor's advice for such a matter is too expensive. Unfortunately, pharmacists have been found to score less than 50% on skin cancer knowledge questionnaires [16]. Thus, it may be prudent to implement health promotion campaigns that seek not only to increase skin cancer knowledge in rural and impoverished communities, but also to raise the educational standards for pharmacists and other allied healthcare workers.

Perceived threat and self-efficacy were routinely found to be important predictors of health behavior change. Educational initiatives would raise awareness of the risk of skin cancers and emphasize that risk can be effectively reduced if skin protection methods are utilized. Studies indicate that knowledge of skin cancers does not always correlate with adoption of protective behaviors, suggesting a need to address this particular divide [21]. A lack in the availability of personnel and material, cost, inconvenience, and a desire for a tanned appearance are reasons given for the disconnect between education and implementation of desired attitudes [21, 22]. Remedies proposed include easy access to sunscreen dispensers in the workplace and promotion by respected community members both acting as tangible reminders of the value of skin protection. In the United States, farm workers are trained to educate and promote healthy habits among their coworkers through the* “Promotora de Salud” *(Health Promoters) program [34, 35]. A similar program could feasibly be adapted in Iran, where health promoters are considered trusted and respected peers. By increasing available resources, threat perception, and self-efficacy, significant changes in prevention behaviors can occur, especially in a changing social environment.

It is also important to consider which methods of skin protection are preferred for each population subgroup. Previously accepted practices should also be considered when attempting to improve health behaviors. For example, in the case of rural farmers, avoiding sunlight may not be an option, as they must work the fields and do not have the option to follow the best practices such as seeking shade during peak sun times. However, farmers and other outdoor laborers were likely to wear small hats as their primary means for sun protection, even though they show little to no evidence of skin cancer prevention. In contrast, evidence-based practice of using wide-brimmed hats demonstrably showed a protective effect in the prevention of skin cancers. In* Operation Hat Check, *small hats were exchanged with those having wide brims, thus providing the adequate protection needed from harmful rays that could cause skin cancer [36].

Studies found that children are more vulnerable to the harmful effects of prolonged sun exposure than adults and that most UVR absorption occurs before the age of eighteen [13]. School educational programs on the use of proper skin protection practices should be implanted at an early age when these important life-saving habits can be instilled into children. Such interventions should also be extended to teachers and parents. According to one study, nearly 50% of Iranian secondary school teachers did not have the appropriate levels of knowledge concerning skin cancers [12].

## 5. Limitations

Limitations to this literature review exist and are acknowledged. Although searches were performed in several databases, it is always possible that we did not identify all of the published studies related to the knowledge, attitudes, beliefs, and practices associated with preventing skin cancer among the Iranian population. Articles from grey literature were not included, markedly reducing the number of studies cited. We also limited the generalizability of our findings by not considering self-reported data of the included studies due to increased probabilities of recall errors and social desirability biases. However, previous studies have validated self-report of sun protection strategies and found it to be as good as objective measures [37, 38].

## 6. Conclusion

In light of the increasing trends of skin cancer incidence rates among Iranians, there is an urgent need to continue exploring knowledge, attitudes, and practices of skin cancer prevention. This is especially important for those Iranians who are at a heightened risk. The findings of this study show that efforts to prevent skin cancers are needed. A lack of knowledge coupled with a low sense of urgency among Iranians towards their perceived susceptibility and the severity of skin cancers gives relevance to these findings. This is exacerbated by the Iranians' low usage of skin cancer prevention practices. Promoting access to and delivery of skin cancer prevention services can lead to increased protection, which may ultimately reduce the mortality rates associated with skin cancers, the most commonly diagnosed cancer in Iran.

## Figures and Tables

**Table 1:** Summary of the included studies.

First author, date, location	Data collection method, sample size (*n*), gender, age	Knowledge, attitudes, and beliefs	Skin cancer prevention practice
Firooz, 2007, Tehran [9]	Interview/questionnaire, *n* = 250 (patients), 61% males, mean age: 35.9 years	—	Sunscreen: never (87.6%), sometimes (5.6%), often (4.0%), and always (2.8%) Sunshade usage (cap, etc.): never (86%), sometimes (7.6%), often (4.8%), and always (1.6%) Daily direct sun exposure: <1 hr (30%), 1–4 h (60.8%), and >5 h (9.2%)

Davati, 2008, Yazd [10]	Questionnaire, *n* = 256 (clinic attendees), 100% females, mean age: 38.2 years	Obstacles to sunscreen use: expensive: 56%, side effects: 34%, change in skin appearance: 29%, not effective: 17.7%, luxury item: 17%, governmental laws and regulations: 8.5% Obstacles to use of other sun protective items: cultural-religious beliefs: 62.6%, luxury: 42%, expensive: 23%, not effective: 13.9%, governmental laws and regulations: 9.2%	Sunscreen: 15.8% Hats: 3.4% Gloves: 3.4% Sunglasses: 11.6% Limit spending time outdoor: 53% Seeking shades: 94%

Maleki, 2008, Mashhad [11]	Questionnaire, *n* = 802 (university students), 51% females, age range: 20–25 years	45% correct answers to 75% of questions; 92.3% knew that sunlight protection practices are important in skin cancer prevention, and 57% knew sunscreen creams shall be used during all seasons	75% no protection behavior

Mazloomy-Mahmoodabad, 2010, Yazd [12]	Questionnaire, *n* = 440 (secondary school teachers), 61% females, mean age: 37.98 years	Knowledge (possible range: 0–49): total mean = 24.20 (male mean = 24.05; female mean = 24.29) Attitude (possible range: 13–39): total mean = 28.10 (male mean = 27.74; female mean = 28.34)	Performance (possible range: 0–16): total mean = 6.35 (male mean = 5.23; female mean = 7.07) Sunscreen: 56.8% Clothing: 38.2% Masked hats: 10.2% Gloves: 12.3% Sunglasses: 27.5% Used none of the above measures: 17%

Baghianimoghadam, 2011, Yazd [13]	Questionnaire, *n* = 360 (high school students: 180 cases and 180 controls), 100% females, mean age: 16.04 years	Perceived susceptibility (possible range: 4–20): case 12.77 and control 13.11; perceived threat (possible range: 5–25): case 17.71 and control 18.04; rewards (possible range: 4–20): case 10.59 and control 9.86; threat appraisal (possible range: 5–25): case −19.81 and control −20.22; fear (possible range: 5–25): case 17.18 and control 16.94; self-efficacy (possible range: 4–20): case 14.83 and control 14.98; response efficacy (possible range: 6–30): case 21.63 and control 21.58; response costs (possible range: 5–25): case 15.25 and control 14.06; coping appraisal (possible range: 5–25): case 21.13 and control 22.49; protection motivation (possible range: 7–35): case 27.11 and control 27.05	Behavior (possible range: 0–12): case 3.92 and control 4.04

Mazloomy-Mahmoodabad, 2011, Yazd [14]	Questionnaire, *n* = 230 (university students), 72.2% females, 27.8% males, mean age: 21 years	Knowledge (possible range: 0–49): total mean = 25.98 (male mean = 27.65, female mean = 25.72, medical students mean = 27.70, and nonmedical students mean = 21.89) Attitude (possible range: 13–39): total mean = 30.83 (male mean = 30.31, female mean = 29.04, medical students mean = 31.38, and nonmedical students mean = 29.85)	Performance (possible range: 0–16): total mean = 7.67 (male mean = 8, female mean = 6.31, medical students mean = 8.06, and nonmedical students mean = 6.06) Avoid sun exposure: 77.8% Sunscreen use: 60% Clothing: 40.4% Masked hats: 13% Gloves: 9.6% Sunglasses: 19.6%

Mousavi, 2011, Tehran [15]	Questionnaire, *n* = 400 (general population), 48% females, mean age: 34.7 years	90.8% knew that they should protect their skin against sunlight; 89.5% more exposure = more harm; 87.2% knowledge of adverse effects; 80.5% exposure harmful in children; 68.8% tanning harmful; 58.8% sunscreen not an easy task; 61.8%, skin cancer can be prevented; 9.5%, cancer risk is highly probable; 78.5%, tanning makes skin look charming	Sunscreen: 31.8% Clothing: 41% Sunbathed: 88.8% (<5 times/year) Never tanned/tanned only once: 87.8%

Movaffagh, 2011, Mashhad [16]	Questionnaire, *n* = 76 (pharmacists), 52.6% females, mean age (males): 33 years, mean age (females): 36.25 years	67.5% knew the correct application time of sunscreen and 15% knew the SPF definition perfectly	Sunscreen: 68.8% (72.7% females/27.3% males) Sunscreen correctly: 12.5% (every 2-3 hours) Sunscreen just in morning and noon: 26.3% Other times: 28.8% No sunscreen: 31.2% Sunscreen just in summer: 12.5% Sunscreen sometimes: 10% Sunscreen all seasons: 38.8%

Mirzaee, 2011, Yazd [17]	Questionnaire, *n* = 165 (mothers of children aged 1–8: 75 in interventional group and 90 in control group), intervention mean age: 30.82 years, control mean age: 31.15 years	Knowledge Intervention: 4.80; control: 4.85 Self-efficacy Intervention: 20.90; control: 21.21 Barriers Intervention: 10.06; control: 10.05 Norms Intervention: 31.84; control: 30.26 Expectations Intervention: 33.68; control: 32.50	Behaviors Intervention: 5.10; control: 5.15

Askarian, 2013, Shiraz [18]	Questionnaire, *n* = 400 (medical students), 56.2% females, mean age: 21.5 years	—	Sunscreen: total = 52% (80.4% females and 13.5% males)

Changizi, 2013, Tehran [19]	Questionnaire, *n* = 620 (general population), 67% females	5.85 are aware of their family history of skin cancer; 57.56% knew about increased risk of skin cancer by using tan machines; 30.32% use different tools for tanning	Sunscreen: 16.67% Sunglasses: 45.83% 24 use solarium for tanning

Davati, 2013, Tehran [20]	Questionnaire, *n* = 941 (female high school students)	Knowledge regarding degree of SPF: SPF 15 (3.2), SPF 20 (3.1), SPF 25 (7.7), SPF 30 (24.4), 30 < SPF < 50 (15.9), SPF > 50 (8.8), and do not know (36.4) Sunscreen cream choice: did not know (39.4), no need to renew (21.8), better to renew every 5 hours (16.6), and better to renew every 2 hours (22.2) Timing for sunscreen: did not know (26.1), half an hour before exiting home (30.6), right before exiting home (26.5), and 10 minutes before exiting home (16.8) How much sunscreen applied each time: no specific amount (52.2), more than one knuckle (7.9), one knuckle (20.4), and less than a knuckle (19.6) Sun avoidance during high sunlight hours: perceived severity (practicing: 17.57; not practicing: 16.93) Walking in shaded or covered places: perceived sensitivity (practicing: 8.07; not practicing: 7.49) and perceived severity (practicing: 17.48; not practicing: 16.22) Sunscreen: perceived sensitivity (practicing: 8.32; not practicing: 7.79), perceived severity (practicing: 17.74; not practicing: 16.97), and perceived benefit (practicing: 11.39; not practicing: 10.8) Gloves: perceived sensitivity (practicing: 6.77; not practicing: 7.96), perceived severity (practicing: 15.58; not practicing: 17.2200 ± 4.4), and perceived benefit (practicing: 9.58; not practicing: 11.00) Sunglasses: perceived sensitivity (practicing: 8.28; not practicing: 7.76) and perceived severity (practicing: 17.72; not practicing: 16.90)	Sun avoidance during high sunlight hours: yes (36.1), no (47.1), and not possible (16.8) Walking in shaded or covered places: yes (74.6), no (16.7), and not possible (8.7) Sunscreen use: yes (24.7), sometimes (47.4), and no (27.9) Cap or veil use: yes (10.3), sometimes (29.2), and no (60.5) Gloves: yes (12.6), sometimes (12.6), and no (83.5) Sunglass use: yes (32.2), sometimes (36.6), and no (31.2)

Ramezanpour, 2013, Zanjan [21]	Questionnaire, *n* = 518 (259 hospital personnel and medical and nursing students (case) and 259 laypeople (control)), case: 94.2% females, control: 78.8% females, mean age: 28.68 years	Sunscreen use knowledge (possible range: 0–11): case 7.17 and control 5.60 Attitude towards sunscreen use (possible range: 11–55): case 42.13 and control 41.64	Sunscreen: case (76.8%), control (71%), male (34.3%), and female (80.1%) Physician (85.7%), nurse (78.7%), student (77.5%), midwife (93.3%), lab tech (82.4%), and nursing assistant (44%)

Tazval, 2013, Ilam [22]	Questionnaire, *n* = 248 (male farmers), mean age: 42.61 years	14.5%, high perceived vulnerability; 30.6%, high perceived severity; 15.7%, low rewards (extrinsic/ intrinsic) from unprotected behaviors; 60.1%, unacceptable threat appraisal; 39.1%, acceptable threat appraisal	—

Golpour, 2014, Sari [23]	Questionnaire, *n* = 2000 (middle school and high school students), 64% males, mean age: 15.07 years	Male: 36.99% good knowledge, 58.28% moderate knowledge, 29.3% low knowledge, and 1.41% high knowledge Female: 20.2% low knowledge, 32.46% medium knowledge, 56.53% good knowledge, and 8.8% high knowledge	Sunblock gels: 38.32% females and 14.61% males Sunglasses: 35.21% females and 9.58% males

Nadrian, 2014, Yazd [24]	Questionnaire, *n* = 75 (university students), 100% females, mean age: 20.7 years	Knowledge: 58.8% Perception: 66.1% Stimulant factors: 60.7%	Sunscreen: 65.3% Long-sleeved shirts: 37.3% Hats: 16% Gloves: 14.7% Sunglasses: 25.3% (not regularly) Spending time outdoor after maximum sun exposure: 83% Used none of the above measures: 6.7% Skin cancer prevention behavior: 48%

Sadeghi, 2014, Kerman [25]	Interview/questionnaire, *n* = 200 (farmers, 100 in intervention group and 100 in control group), intervention: 79 males, control: 77 males, intervention mean age: 47.92 years, control mean age: 48.42 years	Knowledge Intervention: 13.14; control: 12.94 Perceived sensitivity Intervention: 31.82; control: 30.77 Perceived force Intervention: 33.94; control: 32.64 Perceived benefits Intervention: 33.19; control: 32.71 Perceived barriers Intervention: 33.30; control: 21.58 Guide for action Intervention: 13.72; control: 13.08 Self-efficacy Intervention: 29.53; control: 29.31	—

Tabatabayian, 2014, Isfahan [26]	Questionnaire, *n* = 1139 (high school students), 55.4% males, mean age: 16 years	53% knew about harms of sunlight; 31.5% had basic knowledge about skin cancer; 68.5% knew about sun protection methods	Sunlight protection tools: 27.5%

Dehbari, 2015, Tehran [27]	Questionnaire, *n* = 201 (university students), 100% females, mean age: 24.49 years	—	Sunscreen use: never (11.9%), sometimes (27.4%), often (20.4%), and always (40.3%) Wearing a hat: never (55.2%), sometimes (32.8%), often (10.9%), and always (1%) Gloves: never (72.1%), sometimes (15.4%), often (10%), and always (3.5%) Sunglasses: never (20.9%), sometimes (36.3%), often (26.9%), and always (15.9%)

Hoseini, 2015, Zahedan [28]	Questionnaire, *n* = 200 (sixth-grade students), 100% males	Knowledge: intervention 9.55% and control 9.7%	Intervention Sunscreen (during sun exposure): 36% Sunscreen reapplication (after washing hands and face): 19% Long-sleeved shirts: 10% Hats: 21% Sunglasses: 31% (not regularly) Spending time outdoor during high sun exposure: 9% Seeking shades: 48% Intervention: 6.15; control: 6.22

Morowati-Sharifabad, 2015, Kazeroon [29]	Questionnaire, *n* = 300 (farmers), 75% males, mean age: 47 years	72.7%, knowledge about sunlight and skin cancer	Sunscreen: 9.7% Long-sleeved shirts: 52% Hats: 31.7% Gloves: 7.3% Sunglasses: 12%

Afshari, 2016, Tuyserkan [30]	Questionnaire, *n* = 200 (farmers), age range: 18–60 years		Sunscreen: 31.5% Long-sleeved shirts: 65% Hats: 53.5% Gloves: 3% Sunglasses: 19% (not regularly)

Babazadeh, 2016, Chaldoran County [31]	Questionnaire, *n* = 238 (farmers), 100% males, mean age: 35.5 years	Perceived susceptibility: 15.45%, perceived severity: 36.71%, rewards: 32.22%, response efficacy: 29.03%, protection motivation: 21.56%, response cost: 19.57%, self-efficacy: 23.15%	Skin cancer preventive behaviors: 21.68%

Zareban, 2016, Zahedan [32]	Questionnaire, *n* = 240 (high school female students, 120 in intervention group and 120 in control group), intervention mean age: 16.14 years, control mean age: 16.16 years	Knowledge Intervention: 6.4; control: 6.01 Perception Intervention: 23.05; control: 24.12 Stimulants Intervention: 4.14; control: 3.99 Norms Intervention: 2.31; control: 2.38	Behaviors: Intervention: 3.01; control: 3.1
